# Correction: A prospective cohort study of dynamic cell-free DNA elevation during cardiac surgery with cardiopulmonary bypass

**DOI:** 10.1371/journal.pone.0316868

**Published:** 2024-12-30

**Authors:** Shlomo Yaron Ishay, Muhammad Abu-Tailakh, Lior Raichel, Tal F. Hershenhoren, Menahem Matsa, Oren Lev-Ran, Sahar Gideon, Amos Douvdevani

There is an error in affiliation 2 for Muhammad Abu-Tailakh. The correct affiliation 2 is: Recanati School for Community Health Professions, Department of Nursing, Faculty of Health Sciences, Ben-Gurion University of the Negev, Beer-Sheva, Israel and Soroka University Medical Center, Beer-Sheva, Israel.
