# Supplementary material for: A prospective cohort study of dynamic cell-free DNA elevation during cardiac surgery with cardiopulmonary bypass
Source: PLoS One. 2022 Oct 27;17(10):e0276443. doi: 10.1371/journal.pone.0276443 (PMC9612555; doi:10.1371/journal.pone.0276443)
Supplement: Attachment — Submitted filename: Response to Reviewers.docx [file pone.0276443.s001.docx]

Wednesday, 6 July 2022

## Re: PONE-D-22-11026

***A prospective cohort study of dynamic cell-free DNA elevation during cardiac surgery with cardiopulmonary bypass***

Dear Dr. Johnson

Please find bellow the detailed Point by point reply to remarks of academic editor and reviewers.

## **Academic Editor’s Comments**

Please ensure that your manuscript meets PLOS ONE's style requirements, including those for file naming. The PLOS ONE style templates can be found at 
[https://journals.plos.org/plosone/s/file?id=wjVg/PLOSOne_formatting_sample_main_body.pdf](https://journals.plos.org/plosone/s/file?id=wjVg/PLOSOne_formatting_sample_main_body.pdf) and 
[https://journals.plos.org/plosone/s/file?id=ba62/PLOSOne_formatting_sample_title_authors_affiliations.pdf](https://journals.plos.org/plosone/s/file?id=ba62/PLOSOne_formatting_sample_title_authors_affiliations.pdf)

Reply: The revised manuscript is edited according to PLOS ONE's style requirements

2. Please provide additional details regarding participant consent. In the ethics statement in the Methods and online submission information, please ensure that you have specified what type you obtained (for instance, written or verbal, and if verbal, how it was documented and witnessed). If your study included minors, state whether you obtained consent from parents or guardians. If the need for consent was waived by the ethics committee, please include this information.

Reply: According to editor’s instructions we provide more details regarding participant consent in Methods. ” Following explanation from authorized personnel, all participants gave their written informed consent per the Helsinki Declaration. According to regulations, signed forms are saved in the study folder. The study was carried out under relevant guidelines and rules.”

We had no minors in the study, forms were signed only by consent adults.

3. We note that the grant information you provided in the ‘Funding Information’ and ‘Financial Disclosure’ sections do not match.

When you resubmit, please ensure that you provide the correct grant numbers for the awards you received for your study in the ‘Funding Information’ section.

4. Thank you for stating the following in the Acknowledgments Section of your manuscript:

"This work was supported by the Dr. Montague Robin Fleisher Kidney Transplant Unit Fund."

We note that you have provided funding information that is not currently declared in your Funding Statement. However, funding information should not appear in the Acknowledgments section or other areas of your manuscript. We will only publish funding information present in the Funding Statement section of the online submission form. 
Please remove any funding-related text from the manuscript and let us know how you would like to update your Funding Statement. Currently, your Funding Statement reads as follows:

"No"

Please include your amended statements within your cover letter; we will change the online submission form on your behalf.

Reply: We have deleted the Funding Statement from the manuscript. In the Funding Statement section of the online submission form there is no option to insert our financial support. Please include our statement: "This work was supported by the Dr. Montague Robin Fleisher Kidney Transplant Unit Fund." We have included this request in the Cover letter.

5. Thank you for stating the following in your Competing Interests section:

"NO authors have competing interests"

Please complete your Competing Interests on the online submission form to state any Competing Interests. If you have no competing interests, please state "The authors have declared that no competing interests exist.", as detailed online in our guide for authors at [http://journals.plos.org/plosone/s/submit-now](http://journals.plos.org/plosone/s/submit-now) 

This information should be included in your cover letter; we will change the online submission form on your behalf.

Reply: Following this remark, in the cover letter, we have requested to change the statement to "The authors have declared that no competing interests exist."

6. We note that you have indicated that data from this study are available upon request. PLOS only allows data to be available upon request if there are legal or ethical restrictions on sharing data publicly. For more information on unacceptable data access restrictions, please see [http://journals.plos.org/plosone/s/data-availability#loc-unacceptable-data-access-restrictions](http://journals.plos.org/plosone/s/data-availability#loc-unacceptable-data-access-restrictions).

In your revised cover letter, please address the following prompts:

a) If there are ethical or legal restrictions on sharing a de-identified data set, please explain them in detail (e.g., data contain potentially sensitive information, data are owned by a third-party organization, etc.) and who has imposed them (e.g., an ethics committee). Please also provide contact information for a data access committee, ethics committee, or other institutional body to which data requests may be sent.

Reply: We have no restriction, and we agree to publish the anonymized data set of our study

b) If there are no restrictions, please upload the minimal anonymized data set necessary to replicate your study findings as either Supporting Information files or to a stable, public repository and provide us with the relevant URLs, DOIs, or accession numbers. For a list of acceptable repositories, please see [http://journals.plos.org/plosone/s/data-availability#loc-recommended-repositories](http://journals.plos.org/plosone/s/data-availability#loc-recommended-repositories).

Reply: Data was uploaded

We will update your Data Availability statement on your behalf to reflect the information you provide.

## Reviewers #1' comments:

## It is noteworthy that plasma cfDNA levels do not solely reflect NETosis but may rather arise from the damaged tissue (e.g. endothelial injury, cardiac injury). Thus, higher neutrophil numbers might be due to an increased inflammatory response.

This aspect should be considered by the authors. Hence, the conclusion in the abstract section (lines 38-39) is not supported by the data in the manuscript, as the authors did not distinguish between cfDNA and NETs.

Reply: According to the reviewer’s remark, this conclusion in the abstract was deleted.

- Line 55: NETs generally induce thrombosis and endothelial injury, independent of degradation

Reply: The sentence was rewritten “When degraded, NETs are a source of circulating cfDNA and histones and act together as DAMPs to contribute to inflammation [8], thrombosis [9], and endothelial injury [10]”

- Detailed description of the method used to quantifiy cfDNA should be included in the Methods section on page 5, including the type of anticoagulant used, centrifugation steps, Sybr gold concentration etc. Please also include manufacturers’ name.
- Which DNA standard was used in this study? Range of DNA standard?

Reply: As requested by the reviewer, cfDNA measurement method is now described in detail. No anticoagulation was used. As described, serum from coagulated blood was used to test cfDNA.

- Include the type of statistical test in all figure captions

Reply: as suggested, statistical tests are now described in figure captions

- Lines 175-176: did patients with PVD have lower baseline cfDNA levels? If so, the authors should include this information in the manuscript

The baseline cfDNA of patients with PVD was not lower than the baseline of other patients (p=0.771).

- Did patients with high delta cfDNA40-20 levels (Fig1B) have higher cfDNA levels at baseline?

Reply: As shown in revised Fig 1B, baseline cfDNA was not higher in patient with high delta cfDNA40-20 levels. Kruskal-Wallis test, baseline of three groups (p=0.539).

Please include patients’ cfDNA baseline levels in the individual groups (Fig 1B). Are there any statistical differences at the time point 20 min CPB between the different groups? It looks like the cfDNA levels in the first group are markedly elevated when compared to the groups of patients with longer ICU stays (Fig 1B).

Reply: Following the reviewer’s remarks, we modified Fig 1B to include baselines and to show comparisons between groups. By the Kruskal-Wallis test, baseline (p=0.539) and 20 min (p=0.190) are not different between groups.

Same for the time point 40 min.

Reply: By Kruskal-Wallis test and Dunn’s comparison for the 40 min points, the <18hr group differed from the >48hr group (p=0.018).

- In fact, high delta cfDNA40-20 negatively correlates with ICU / hospital LOS.

How do the authors explain that? The assumption (lines 254-255) that inverted correlation of delta cfDNA40-20 and neutrophils with LOS may be explained by the elimination of NETing neutrophils from the circulation due to microvascular obstruction is very speculative and not satisfactory.,

Reply: Following the reviewer’s remarks, we have modified the Discussion to explain in more details with new references, two possible mechanisms for the inverse correlation of ΔcfDNA 40-20 with outcomes.

“*However, unlike what we expected, the dynamic changes in cfDNA levels (ΔcfDNA 40-20) were lower in patients with worse outcomes, including prolonged ventilation time and prolonged LOS in ICU and hospital. Surgery time may be too short for damaged tissue to release a considerable amount of cfDNA, and what we observe in this short time frame is primarily released from fast-reacting neutrophils. As in SIRS associated with sepsis or trauma [**18**,* *19**], the primary source of cfDNA in inflammation induced by CPB could be NETs released from activated neutrophils that undergo NETosis [**20-23**]. Support for this possibility is the correlation of neutrophil number at the end of the surgery with ΔcfDNA 40-20. Low neutrophils number is associated with reduced elevation of cfDNA. Thus, it is possible that in patients with complications, the inverse correlation of ΔcfDNA 40-20 with outcomes is caused by impaired neutrophils that fail to recruit and release less cfDNA.*

*An additional conceivable explanation for the inverse correlation of ΔcfDNA 40-20 with outcomes is derived from the inverse correlation of ΔcfDNA 40-20 with PVD and the association of PVD with outcomes. As for inferior outcomes, patients with PVD had lower ΔcfDNA 40-20 than those without PVD. It is well-known that vascular health affects outcomes [**25**]: In our study, a third (33%) of the patients with prolonged ICU-LOS suffered from PVD, while in patients with shorter LOS, the PVD rate was only 9.6%. In CPB, it was demonstrated that despite full blood heparinization, consumptive coagulopathy related to ongoing intrinsic and extrinsic system activation and thrombin generation continues at low levels [**27**]. Endothelial dysfunction is associated with pro-coagulation [**25**], perhaps the worse outcome of patients with PVD is related to uncontrolled coagulation driven by proinflammatory cytokines and DAMPs. Coagulation in patients with vascular disease may lock neutrophils in the microvasculature and reduce cfDNA released into circulation. This suggested mechanism is attractive since it explains in a patient with complications, bleeding due to consumptive coagulopathy, and the damage to organs by capillary obstruction. If this mechanism is valid, reduced changes in cfDNA during CPB indicate an elevated risk for blood loss and organ dysfunction.“*

Does a strong initial inflammatory response (reflected by high neutrophil counts, high cfDNA levels) support patients’ recovery?

Reply: Elevated cfDNA is usually associated with worse outcomes. It is challenging to explain the support of recovery by elevated cfDNA. However, we cannot exclude that patients with worse outcomes have impaired neutrophils, causing poor neutrophil recruitment, reduced NETs production, and minor cfDNA released into circulation.

In view of the reviewer’s remark, we included this mechanism in the revised discussion.

Did the authors measure additional inflammatory markers like IL-6?

Reply: Unfortunately, measurement of inflammatory mediators like CRP or IL-6 were not part of this study protocol, and samples are not available anymore for measurement.

As already mentioned, it is mandatory to prove if 1. cfDNA levels differ at baseline between groups and 2. cfDNA levels at 20 min and also at 40 min are different between groups (Fig 1 B)

Reply: Following the reviewer’s remarks, we have modified Fig 1B to show the comparisons between groups. cfDNA at baseline were similar. Neither comparison of the groups at 20 minutes showed significance. We observed significant changes between two 40 minutes groups (<18 hours vs >48hours).

- Although this study did not aim to explain the mechanism behind these findings, I strongly recommend to include additional data showing dynamics of at least one inflammatory marker (e.g. CRP, IL-6) that may help to interpret these unexpected results.

Reply: Unfortunately, measurement of inflammatory mediators like CRP or IL-6 were not part of this study protocol, and samples are not available anymore for measurement.

## Comments of Reviewer #2

abstract and introduction section) sounds somewhat old-fashioned and part of the referenced bibliography should be replaced with more recent papers.

Reply: Considering the reviewer’s remarks, the Abstract and Introduction were revised with references to more recent papers.

Overall, unfortunately this paper misses the criteria for publication. My points are as follows.
METHODS
The methods section is confusing, and some fundamental clarifications are needed.
The authors present data from a cohort of 71 consecutive elective and low-risk patients who underwent CPB-assisted CABG more than a decade ago.

Can the author clarify the choice of this very specific cohort numerosity

## Reply: Following the reviewer's request, we added a “Sample size considerations” section in Methods:

*“According to preliminary data, we expected cfDNA values before surgery to average around 700±450 ng/ml and to double during bypass. Based on these assumptions and under a type I error probability of 5% (α=0.05), with 70 patients, our study was expected to have a statistical power of 80% to reject the null hypothesis and identify a change of 100% difference in cfDNA levels. “*

and why they are submitting the manuscript more than 10 years after the study execution?

Reply: It is difficult to justify this long delay in publication. We expected that the rise in cell-free DNA during CPB would positively correlate with worse outcomes. A pattern that others and we observed in numerous studies. We were surprised and disappointed by the inverse correlation between the cfDNA levels during CPB at 40 min and early outcomes. Initially, we had no logical explanation for this observation, and we decided to wait a few years for late outcomes. Again, the results of late outcomes did not provide us motivation to publish these results.

Meanwhile, Dr. Ishay, the first author, went for a few years to fellowship, and when he returned, we looked again at the data and observed that the dynamic changes in cfDNA were low in patients with PVD and patients with worse outcomes. These data with new information in the literature on NETosis and coagulation gave us further motivation to write the article.

Regardless of the time it took us to process the data and write the manuscript. We agree with the reviewer and thank him for commenting that our findings “provide insights on the early acute-phase reaction that leads to variable degrees of postoperative systemic inflammatory response in cardiac surgery patients.”

Primary endpoints were:
- Assess the kinetic of cfDNA during CPB;
- Ascertain its association with prolonged ICU and in-hospital stay;
- Evaluate its impact on surgery-related death.
Secondary endpoints were:
- Investigate the association of the cfDNA trajectories with routine lab data;
- Compare the prognostic value of the cfDNA trajectories with standard risk score (STS and EuroSCORE II).

The definition of some outcomes is unclear. Indeed, a prolonged ICU-LOS is defined as >48h (line 84, table 1), but also as >18h (lines 108-109, clinicaltrials.gov) whereas a prolonged in-hospital stay is defined as >15 days (line 85, table 1), but also as >7 days (lines 108-109, clinicaltrials.gov).

Reply: We made some mistakes in the cutoff definition submitted to clinicaltrial.gov. Definition of outcomes used in this study is now under “**Clinical outcomes:”**

Moreover, the mechanical ventilation time is mentioned neither as primary nor as secondary outcome but the extubation protocol was thoroughly described and further analysis was provided.

Reply: Following the reviewer’s remarks we included ventilation time in the primary outcomes.

Furthermore, the section entitled “Criteria for ICU and hospital discharge” did not provide any information about the actual criteria for discharge neither from ICU nor from the surgical ward instead it merely defined what was considered to be normal and prolonged ICU- and hospital-stay.

Reply: According to the reviewer’s remarks we have included in detail the

criteria for ICU and hospital discharge in the Methods section.

Disappointingly, these definitions were incoherent with the rest of the manuscript.

Reply: We repaired this incoherency, definition of outcomes used in this study is now under “**Clinical outcomes:”**

Another major issue is represented by the long-term follow-up (i.e., 54 months) that, in light of the analysis and results provided, sounds very controversial to me, and that basically dampened the modernity of the early-outcome data without providing any relevant information.

Reply: Following the reviewer’s remark we removed the long-term follow-up.

Minor issues:
- Lines 75-76: “SUMC is the sole tertiary medical center in the Negev region” is an irrelevant statement and should be deleted.

Reply: this statement was deleted according to the reviewer’s instruction.

- Line 91: “Ascending aorta-to-right atrial appendage” should be rephrased as “right atrial appendage-to-ascending aorta” to better describe the direction of the extracorporeal circulation.

Reply: the sentence was rephrased according to reviewer’s instruction.

RESULTS
There are also some major areas of concern with this section.
The first concern is related to the choice of the delta-cfDNA 40-20 min. The concentration of cfDNA increased early at CPB-onset, peaked at cross-clamp removal in a time-dependent manner, and returned to baseline 30 minutes after chest closure.

Reply: We tested for correlations of primary and secondary outcomes with cfDNA levels at the various CPB time points. In addition, we correlated outcomes to the dynamic changes in cfDNA (ΔcfDNA) between time points, including cfDNA at 20 minutes minus baseline (ΔcfDNA 20-0), ΔcfDNA 40-20, ΔcfDNA cross-clamp removal -40, ΔcfDNA cross-clamp removal-recovery, ΔcfDNA cross-clamp removal-0 and ΔcfDNA0-recovery. Of these correlations, outcomes correlated best to ΔcfDNA 40-20.

This information is now described in the results.

The authors need to clarify why the delta-cfDNA baseline-to-peak did not undergo analysis whereas the delta-cfDNA 40-20 min was rather chosen.

Reply: among the correlations we tested, we analyzed the correlations of outcomes with the baseline to peak (ΔcfDNA cross-clamp removal-0) and found them inferior to those of ΔcfDNA 40-20.

Furthermore, the results section starts with the description of the cohort characteristics and of the early outcomes. This is absolutely correct.

However, after this preface, the results of the primary outcomes analysis should have been reported first, followed by those of the secondary outcomes. In this sense, the results section appears chaotic, and rephrasing the whole section is mandatory.

Reply: according to the reviewer’s remarks, the Results section was revised intensively.

Primary endpoints:
- The kinetic of cfDNA is well described;
- Its association with the ICU and in-hospital stay is also well described;

- Instead, the analysis of its impact of 30-days surgery-related death in such a small group of very low-risk elective patients is somehow nonsense.

Reply: according to the reviewer’s remark we removed the correlation to 30-days surgery-related death

Secondary endpoints:
- Early postoperative lab data showed the reduction of parameters like hemoglobin, albumin, and calcium (lines 133-134): this phenomenon is trivial, expected, and it is due to several factors such as hemodilution, blood loss, adhesion to the CPB materials (i.e., albumin), and hemostasis, all definitely not being related to cfDNA.

Reply: according to the reviewer’s remark we removed Table 2 of early postoperative lab data

This specific analysis is speculative and perhaps the evaluation of the potential associations of cfDNA levels with those of other common inflammatory markers (e.g., C-reactive protein, lactate dehydrogenase, etc.) would have represented a more consistent point. Indeed, all this was not mentioned in the discussion section. Moreover, the postoperative finding of neutrophilia and lymphopenia is a common occurrence in cardiac surgery patients, and it is considered itself as a marker of inflammatory activation.

Its correlation with the cfDNA trajectory does not implicate a causation (notwithstanding, I understand rational behind the possible explanation that was provided in the discussion section).

Reply: Unfortunately, we have not collected inflammation marker data. We discuss in detail the relationship between cfDNA and neutrophil number.

- The prognostic value of the cfDNA kinetic for the indexed outcomes appears satisfactory as compared to those of the conventional scores. However, it must be noted that these scores were designed and validated to predict early mortality (0% at 30 days), not to predict ICU or in-hospital stay. Hence, this finding can be considered somehow illegitimate.

Reply: We are aware of the limitation of the conventional scores and explain this under Limitations: “*Euroscore and STS scores were designed to predict mortality. Since we had no mortalities, we compared the performance of these scores to predict different outcomes.”*

- The analysis of the predictive performance of the delta-cfDNA 20-40 min for mortality at follow-up was not provided. The low incidence of late mortality is an explanation. However, it is difficult to me to understand the rational behind this investigation given the low-risk cohort and the fact that the hypothetical comparison with prognostic tools calibrated for early mortality would have add almost no information to the manuscript.

Reply: we believe that comparing cfDNA with valid scores gives an essential perspective of its value. Unfortunately, there is no available score to predict ICU and hospital LOS. However, prolonged LOS and mortality share common hazards, and their score could be interchanged with some limitations.

DISCUSSION
As I previously stated, the postoperative inflammatory response is complex to the point that drawing conclusions is a daunting task. Indeed, most of the discussion is based on hypothesis and possible explanations. The only point is that a worse outcome would be expected with a sharp rise in the levels of a DAMP like cfDNA, and this was not the finding of this research. NET-osis and microcirculatory angiopathy are indeed a possible explanation. The correlation with peripheral vascular disease is interesting, but a clear definition of PVD is needed.

Reply: Criteria for PVD are now included in the methods.

However, I think that the finding of this work just shows how the intraoperative activation of immune-inflammatory pathways represents a protective mechanism, that can be maladaptive if dysregulated.

The analysis of this study in the context of the existent evidence is well performed and I agree that a true comparison with the result of other works is unreliable because of the different populations and timeframes. Finally, the limitations section appears adequate.
I offer this criticism with respect and appreciate the authors' time very much.
